# Real and visually-induced body inclination differently affect the perception of object stability

**DOI:** 10.1371/journal.pone.0186431

**Published:** 2017-10-16

**Authors:** Rafael Laboissière, Pierre-Alain Barraud, Corinne Cian

**Affiliations:** 1 Univ. Grenoble Alpes, CNRS, LPNC, Grenoble, France; 2 Univ. Grenoble Alpes, CNRS, TIMC-IMAG, Grenoble, France; 3 Institut de Recherche Biomédicale des Armées, Brétigny sur Orge, France; Tokai University, JAPAN

## Abstract

The prediction of object stability on earth requires the establishment of a perceptual frame of reference based on the direction of gravity. Across three experiments, we measured the critical angle (CA) at which an object appeared equally likely to fall over or right itself. We investigated whether the internal representation of the gravity direction, biased by either simulated tilt (rotating visual surround) or real body tilt situations, influences in a similar fashion the judgment of stability. In the simulated tilt condition, the estimated CA and the perceived gravity are both deviated in the same direction. In the real tilt condition, the orientation of the body affects the perception of gravity direction but has no effect on the estimated CA. Results suggest that people differently weigh gravity direction information provided by visual motion and by visual polarity cues for estimating the stability of objects.

## Introduction

A crucial aspect of the perceptual guidance of motor actions is the ability to predict how visible objects are likely to behave in the near future. People can judge in a quick glance whether an object is likely to return to its vertical upright position or will fall over, what suggests that they have developed a basic understanding of the fundamental physical principles governing the behavior of moving objects. Since an object will fall when its gravity-projected center of mass (COM) lies outside its base of support, therefore a correct estimation of the gravity direction is crucial for assessing the stability of objects. It has been shown that the stability of objects is perceived relative to an internal model of gravity [1, 2]. When observers were tilted s ideways, systematic errors were observed in the direction of the subjective estimate of the visual vertical (i.e; biased toward body orientation [1]). This deviation of the perceived visual vertical when observers are in a roll tilted orientation, also known as the Aubert effect, is probably due to an increased weighting of the body-centered reference frame, which reorients the perceived vertical toward the observer’s body axis [3–5]. This finding suggests that the visual system’s estimates of physical stability incorporate multisensory information and may use biased internal representations of gravity [1].

Moving visual scenes also influence the apparent direction of gravity. Optic flow caused by the relative motion between an observer and the scene may induced a sensation of self-motion referred to as vection. When a stationary observer views a large scene moving uniformly in one direction, he/she soon feels himself moving in an opposite direction while the scene itself may appear to stop moving [6–8]. It was proposed that the occurrence of vection might be linked to the inherent assumption of a stable environment. When observers in upright position are exposed to visual stimuli rotating around the line of sight, vection affects the perceived orientations of the body and the visual objects with respect to gravity [9, 10]. Shortly after initiation of motion, an illusion of continuous motion of the body opposite to the display occurs, which is paradoxical with the sensation of limited body tilt in that direction. This limitation in the sensation of movement is due to the restraining influences from otolith organs, which inform subjects that their body orientation is not changing [6]. The fact that the rotation of the visible surround affects both the visual object and the postural orientations suggests that a shift occurs in the internal representation of the gravity vector [9].

Real body tilt has been shown to induce modifications in the object stability estimation. It is also well known that the vection induced by a rolling visual environment can cause an illusion of body tilt. One might then conclude that a rotating visual scene would have an effect on the judgment of object stability. This would be of prime importance for virtual reality systems, in which vection is used to give the sensation that users are moving while they are actually not. However, the argument above is sound only if the real and the simulated tilt share the same nature and involve the same neuronal and cognitive mechanisms. This is probably not the case, because sensory cues which lead to body orientation perception are different in real and simulated environments. Indeed, real tilt provokes changes in vestibular and somesthetic information, what is not the case for the illusion of tilt induced by motion of the visual field.

The goal of the experiments described in this study is to disentangle the contribution of the different sensory modalities involved in ecological tasks executed in simulated environments. In the first experiment, participants will be required to do two different tasks, namely the estimation of the critical angle of stability of objects and the estimation of the allocentric horizontal direction. These tasks will be performed under two different conditions: either with a real tilt of the body or a induced tilt provoked by a moving circular field. For both cases, the internal representation of the gravity direction must be accessed by the subjects, in order to perform the required estimations. If the representation of the gravity vector is independent of the source of body tilt (either real or simulated), then we should observe similar results in both conditions. Moreover, if the two tasks involve the same type of internal computations, once the gravity direction is determined, then we should observe no interaction effect between the conditions and the tasks.

## Experiment 1

### Methods

#### Participants

The data were obtained from 26 healthy paid volunteers (15 females, 11 males, ages: 18–41 years, mean ± SD: 27 ± 5.7 years). All of them had normal or corrected to normal vision. We screened the subjects by asking them verbally whether they had any past or present vestibular dysfunction. Participants were naïve to the aim of the study and gave their informed and written consent prior to their inclusion in the study. They were free to withdraw from the experiment at any time.

The research was performed in accordance with the ethical standards specified by the 1964 Declaration of Helsinki and approved by the institutional ethics committee of the University Grenoble-Alpes (IRB00010290-2016-09-13).

#### Apparatus

Participants were seated on a chair allowing body roll rotations. The axis of rotation was horizontal and passed approximately through the midpoint between the eyes. The body of the participants was restrained by means of a seat belt and stiff side support surfaces. A headrest fixed on the chair maintained the head aligned with the body. Participants were tested in three body orientations: sitting upright, tilted 30° to the left side, and tilted 30° to the right side. The values of ± 30° for the body tilt were chosen according to results found in previous published studies. For a rotating visual field of 30°/sec, Dichgans et al. found changes in the subjective visual vertical (SVV) direction between 8° and 30° for seven subjects [9], while the three subjects of Held et al. had changes in SVV between 8° and 15° [10]. Body tilt of 30° has been shown to induce deviation of the SVV towards the body Z-axis by amounts varying between 5° and 10°, a phenomenon known as the Aubert effect [11–14]. The chosen value 30° for body tilt should then produce changes in the SVV estimation as in a vection situation where the visual field rotates at 30°/sec (which is the value used in the present experiment, see below).

The visual stimuli were presented in the fronto-parallel plane on a screen with a resolution of 19.4 pixels per degree of visual angle. The screen was placed at a distance of 30 cm from the subject’s eyes. Vision of the screen was constrained by a circular tube (28 cm of diameter), such that the participant could not see anything else beyond the central part of the screen. This avoids the vision of extraneous vertical or horizontal lines in the environment that could bias the responses. The participant’s gaze was aligned with the center of the circular visual field which subtended 50° of visual angle. Observers were tested in a dark room, the only source of light coming from the computer screen.

#### Object stability stimuli

**Fig 1:**
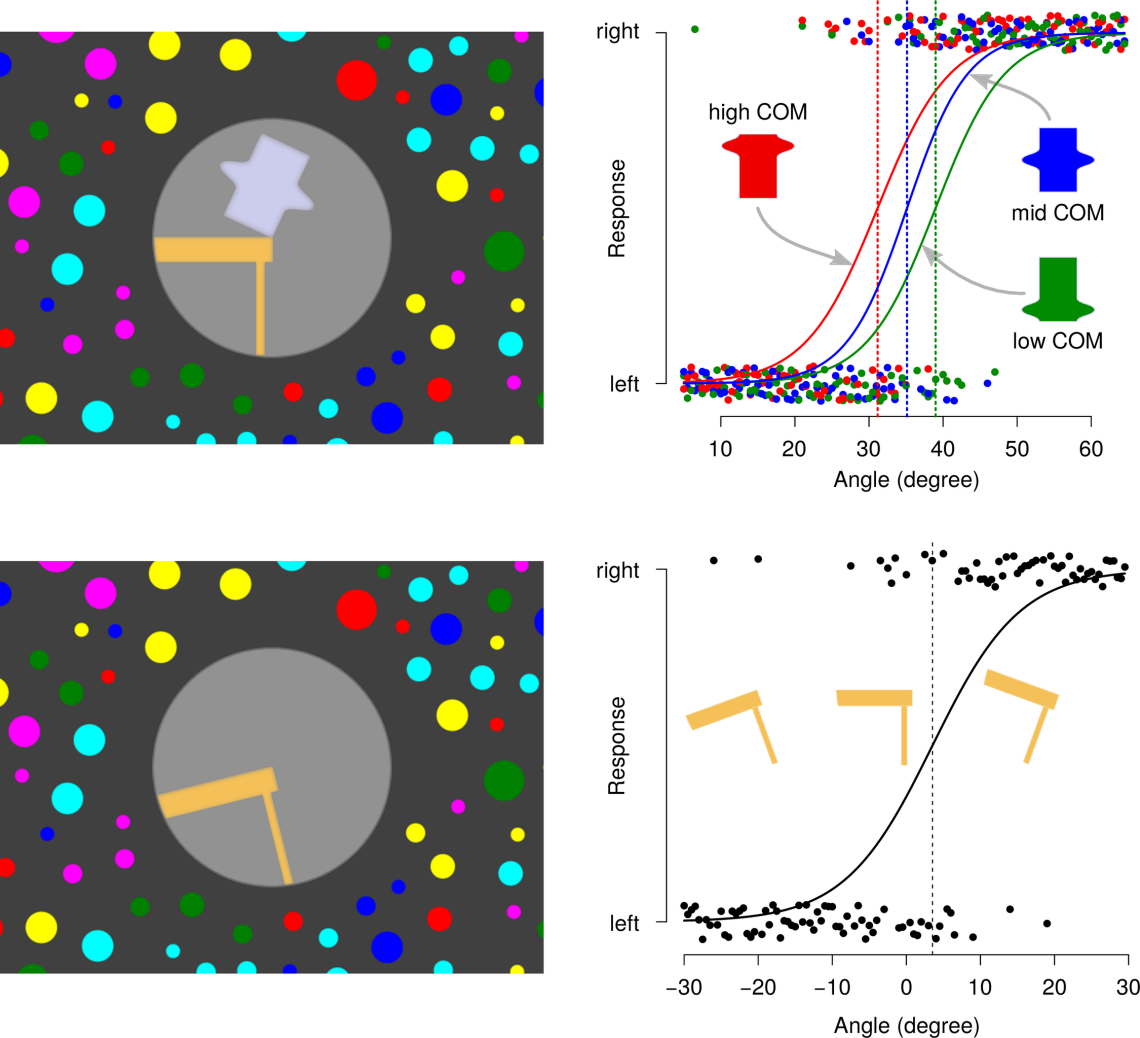
Experimental display and psychometric curve estimation. Examples of the stimuli presented to the subjects are shown in the panels at the left side. Only the central part of the visual scene is represented. Examples of raw data and fitted psychometric curves for representative subjects are shown in the panels at the right side. Stimulus and data for the object stability experiment are shown in the top panels and for the table uprightness in the bottom panels. The vertical axis in the plots represent the response of the subject, either “left” or “right”, indicating the side where he/she thinks the object will fall or to which side the table is tilted. The horizontal axis represent the angle of the support basis of the object with respect to the table (0° means that the object is upright) or the inclination of the upper part of the table (0° means that the table is horizontal, negative/positive angles means that the table is tilted to the left/right, respectively). The dots correspond to the trial responses. Jitter has been added to the vertical position of each point, in order to improve the visualization of the results. The vertical dotted lines indicate the estimated CA, in the object stability task (top), and the estimated TU, in the table uprightness task (bottom).

The visual field was divided in two areas, the center and the surround (see Fig 1). The circular area at the center of the visual field, subtended 15° of visual angle. A two-dimensional scene was presented in this area which contained a table and an object. No 3D hints, like depth or illumination shadows, were presented in the scene. Care was taken to smooth the borders of the object and the table, such that pixelation could not convey hints for detecting the vertical/horizontal screen orientation. The lower right corner of the object and the upper right corner of the table were placed exactly at the center of the scene. This point was also the rotation center for the object. Three objects with the same apparent area but different COM heights were shown (see upper left panel of Fig 1). The object was tilted rightward from 5° to 65° in steps of 0.5°. Each object was then presented in 120 different orientations during each experimental condition, which gives a total of 360 trials. The circular surround was covered with a random pattern of dots of different sizes and colors, covering around 40% of the area. The visual surround either remained static or rotated rightward at an angular velocity of 30°/sec. (See S1 Video for an example of the stimulus display.)

Participants performed the task with either a moving surround (vection-inducing situation) or a static surround. In the moving surround condition, only the body upright condition was tested. In the static surround condition, left, right and upright body orientations were considered. Each condition began with the presentation of the surround and a mask in the central area (grey image and a white fixating dot) during one minute. This amount of time should be long enough to create a vection illusion when the surround is moving [10], even though the existence of the illusion for all subjects is not a strict requirement for the present study. Then, the scene was displayed in the central area during 100 ms and replaced with the mask for 500 ms. The scenes were presented in random order in blocks of 120 trials, with 30 sec of rest between each block. The movement of the dots was continuous during the whole moving-surround sessions. Participants performed a two-alternative forced-choice task to judge whether the object would move to the right side (i.e. fall off the table) or to the left side (i.e. right itself). They responded by pressing the button of joysticks held in the right and left hands using their index finger, respectively.

#### Perceived gravity stimuli

The direction of gravity was indirectly measured using the table uprightness estimation. The same visual surround was used as in the object stability experiment but only the table appeared in the central area (see lower left panel of Fig 1). The table was presented in 120 different orientations, from –30° (rotated counterclockwise) to +30° (rotated clockwise) in steps of 0.5°. The range of tilt was centered about the direction of the gravitational horizontal. Notice that the image of the table used in the stimuli has a polarity, such that the foot could be hardly interpreted as the top and vice-versa, because the top is thicker and has a border going beyond the foot. Moreover, since the tilt of the table is always below 45°, ambiguous interpretations are prevented.

Participants performed the task in the same four conditions as in the object stability estimation task. The same methodology for stimuli presentation was used as in in the object stability experiment. Participants performed a two-alternative forced-choice task to judge whether the table was tilted clockwise (to the right) or counterclockwise (to the left) relative to the horizontal. They responded by pressing the button of joysticks hold in the right and left hands using their index fingers, respectively.

#### Procedure

The experiment was divided in two sessions of one hour each: (1) sitting upright with a static or a moving surround and (2) tilted 30° on the left or 30° on the right with a static surround. In each condition, participants performed first the object stability task and then the table uprightness task. In order to get the participant familiar with the instructions, each task was preceded by 20 stimuli presented with the body in the upright orientation and with a static surround. Half of the participants did session 1 first and the others started with session 2. The order of the conditions during each session was counterbalanced across participants. The two sessions were separated by at least one day.

#### Data analysis

A generalized linear model (GLM) was fitted for the set of 120 trial responses for each participant in each specific experimental condition (and for each COM height, in the case of object stability estimation). The tilt angle of the object or the table was the continuous independent variable in the model. The dichotomous response of the participant was the dependent variable. The error distribution was assumed to be binomial and a logistic function was fitted by using the glmrob function of the R statistical software [15]. This robust regression procedure minimizes the effect of outliers that are clearly response errors. The threshold parameter of the fitted logistic function is the measured value that will be used in the further analysis as the dependent variable. This threshold will be hereafter called critical angle (CA) for object stability task and table uprightness (TU) in the perceived gravity task. Examples of GLM estimation are shown in the right panels of Fig 1.

For each analysis of variance, a linear mixed-effects model (LMM) was fitted to the data by using the lmer function of R [16]. We allowed a deviation of the overall intercept as a random effect for each participant. The height of the COM was considered as a continuous independent variable with values –1, 0, and +1 for the low-, mid-, and high-COM objects, respectively. The orientation of the body was likewise considered as a continuous independent variable, taking the values of –1, 0, and +1 for the left, upright, and right body tilt, respectively. The use of continuous variables presupposes that the effects of the corresponding factors will be linear and this has the advantage of producing a single fitted parameter (the slope), which can be easily interpreted. The nature of the visual surround was a discrete independent variable with two levels (static and rotating). Analyses of variance of type III with Satterthwaite approximation for degrees of freedom were performed through the function anova.merModLmerTest of R [17].

Confidence intervals (CI) for the estimated LMM parameters was done through the function confint.merMod of R. The “profile” method of this function was used, in which a likelihood profile is computed and the appropriate cutoffs are based on the likelihood ratio test. Confidence intervals for model-predicted values were computed by using the bootMer function of R (lme4 package), which performs model-based semi-parametric bootstrap for mixed models. The outcome of the bootstrap procedure is a series of values for the variable of interest (for instance, the predicted value of CA for given combination of conditions), that is expected to follow the posterior distribution of that variable. We ran 1000 simulations for each CI estimation. From those values we extracted the highest density interval that corresponds to 95% of the empirical distribution by using the hdi function of R [18]. This interval is hereafter referred to as the CI.

### Results

**Fig 2:**
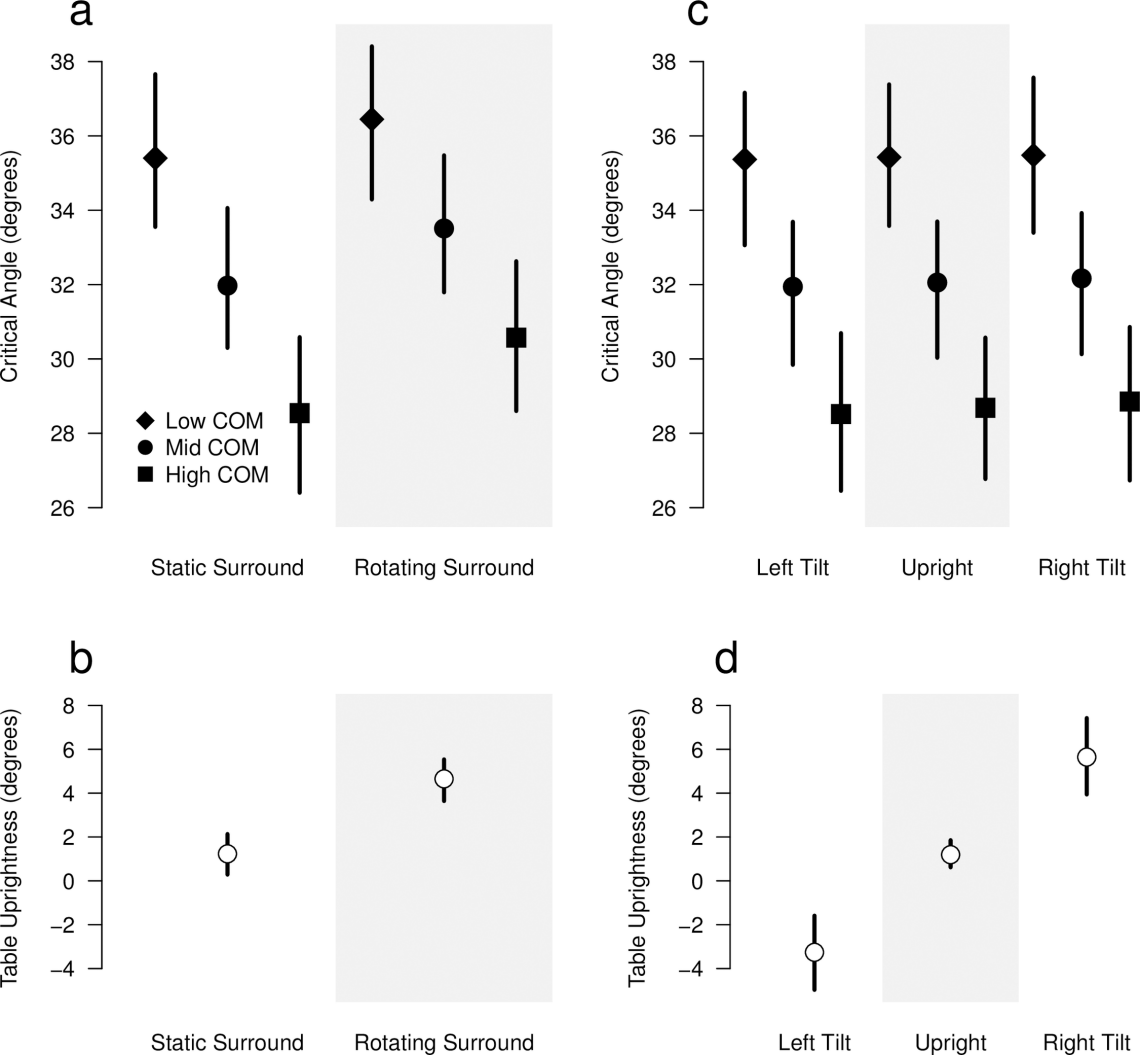
Results of Experiment 1. Mean estimated values of the critical angle (CA) and the table uprightness (TU) are shown with filled and open symbols, respectively. Vertical lines indicate the 95% confidence intervals for each condition. The results of the separated analysis of Exp. 1 data are depicted: CA (panel a) and TU (panel b) for the static vs. rotating background conditions; CA (panel c) and TU (panel d) for the body orientation conditions (30° tilted to the left, upright, and 30° tilted to the right).

Results for the four analyses of variance for Experiment 1 are shown in Fig 2. The movement of the scene surround had an effect on the object stability judgment, the CA being significantly higher for the rotating condition (F(1,127) = 8.96, p < 0.004), see Fig 2A. The difference in CA between the static and the rotating conditions was equal to 1.54° (95% CI [0.54°, 2.54°]). The COM height of the object had a significant effect (F(1,127) = 102.30, p < 0.001) on the CA estimation, with a difference of 3.43° (95% CI [2.56°, 4.30°]) for the low- and high-COM objects in comparison with the mid-COM object (this value is the slope of the linear regression against COM height in the LMM). Low-COM objects had an average CA higher than that for high-COM objects, which is coherent with the prediction from physical principles (see S1 Text). The interaction effect between movement surround and COM height was not significant.

Surround movement also affected the TU judgment (Fig 2B). For our participants, the table had to be tilted clockwise (the same direction of rotation) in order to be perceived as upright. This effect was significant (F(1,25) = 55.03, p < 0.001), with the difference in TU between the static and rotating surround being 3.42° (95% CI [2.50°, 4.34°]).

Real body tilt had no significant effect on CA estimation. However, a significant effect of the COM height was found (F(1,205) = 156.28, p < 0.001), see Fig 2C. It is similar to the one found for the surround movement situation. The difference in CA between the mid- and either the low- or the high-COM objects of 3.37° (95% CI [2.84°, 3.90°]). Real body tilt induced a significant effect on TU estimation (F(1,25) = 28.81, p < 0.001), see Fig 2D. The veridical upright table is perceived as tilted in the opposite direction of the body tilt, with an absolute deviation of 4.45°/side (95% CI [2.80°/side, 6.11°/side]).

**Fig 3:**
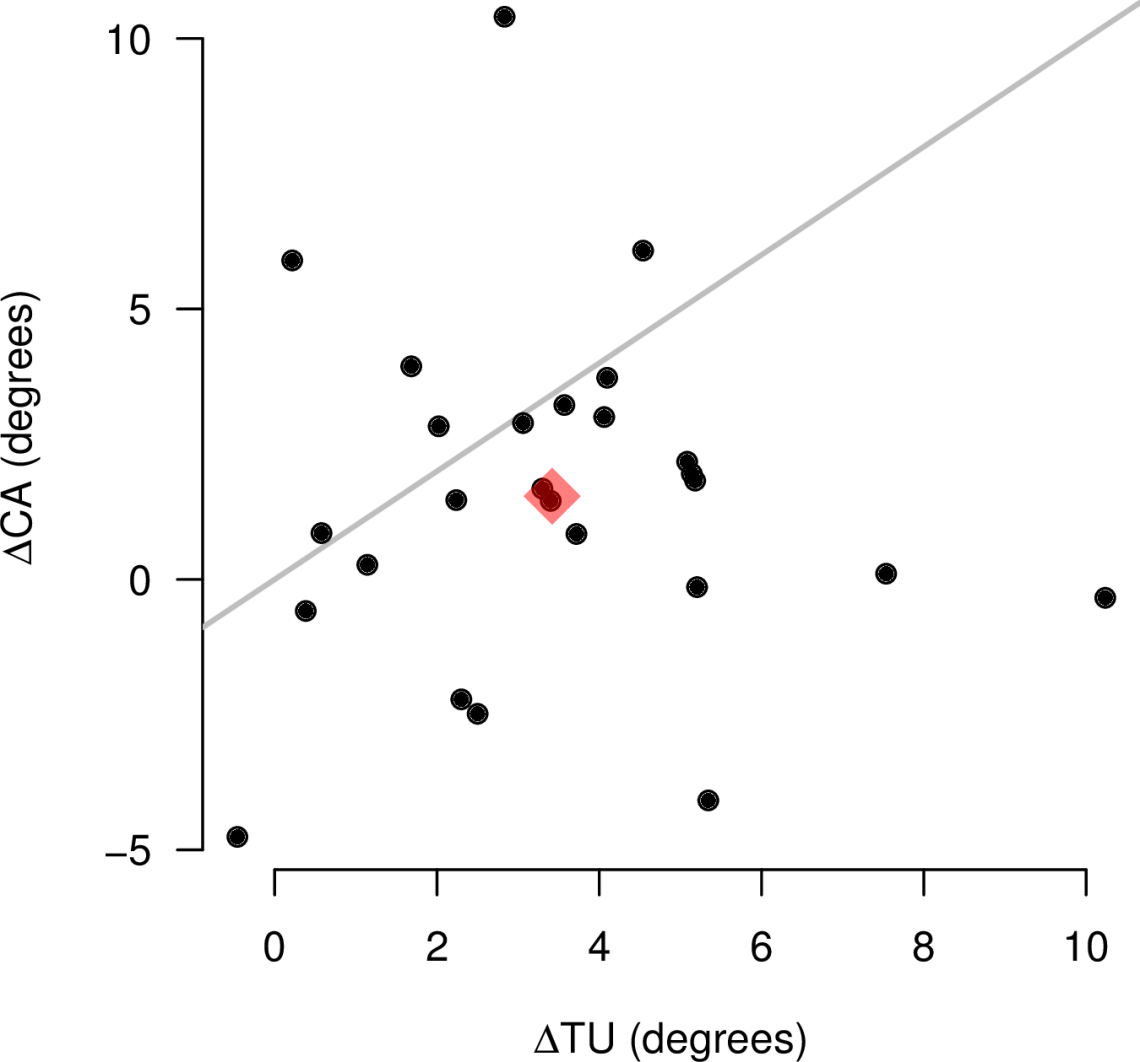
Per-subject differences in angle estimations across background conditions. The difference in the table uprightness (TU) between the rotating and static background is represented in the horizontal axis. The difference in the critical angle (CA) between the rotating and static background, averaged across the three objects, is represented in the vertical axis. The gray line indicates the values for which ΔTU = ΔCA. The red diamond represents the mean population value for both axes.

In the moving surround condition, the average amount of deviation in the estimated CA (1.54°) is smaller than the average deviation in the TU estimation (3.42°). This is illustrated in Fig 3, where both population means are represented by a diamond. In this figure, we also show the individual deviations in the two estimated values for each participant. For 20 out of 26 participants, the deviation in CA (ΔCA) was smaller than the deviation in TU (ΔTU). There is no significant correlation between ΔCA and ΔTU.

## Experiment 2

**Fig 4:**
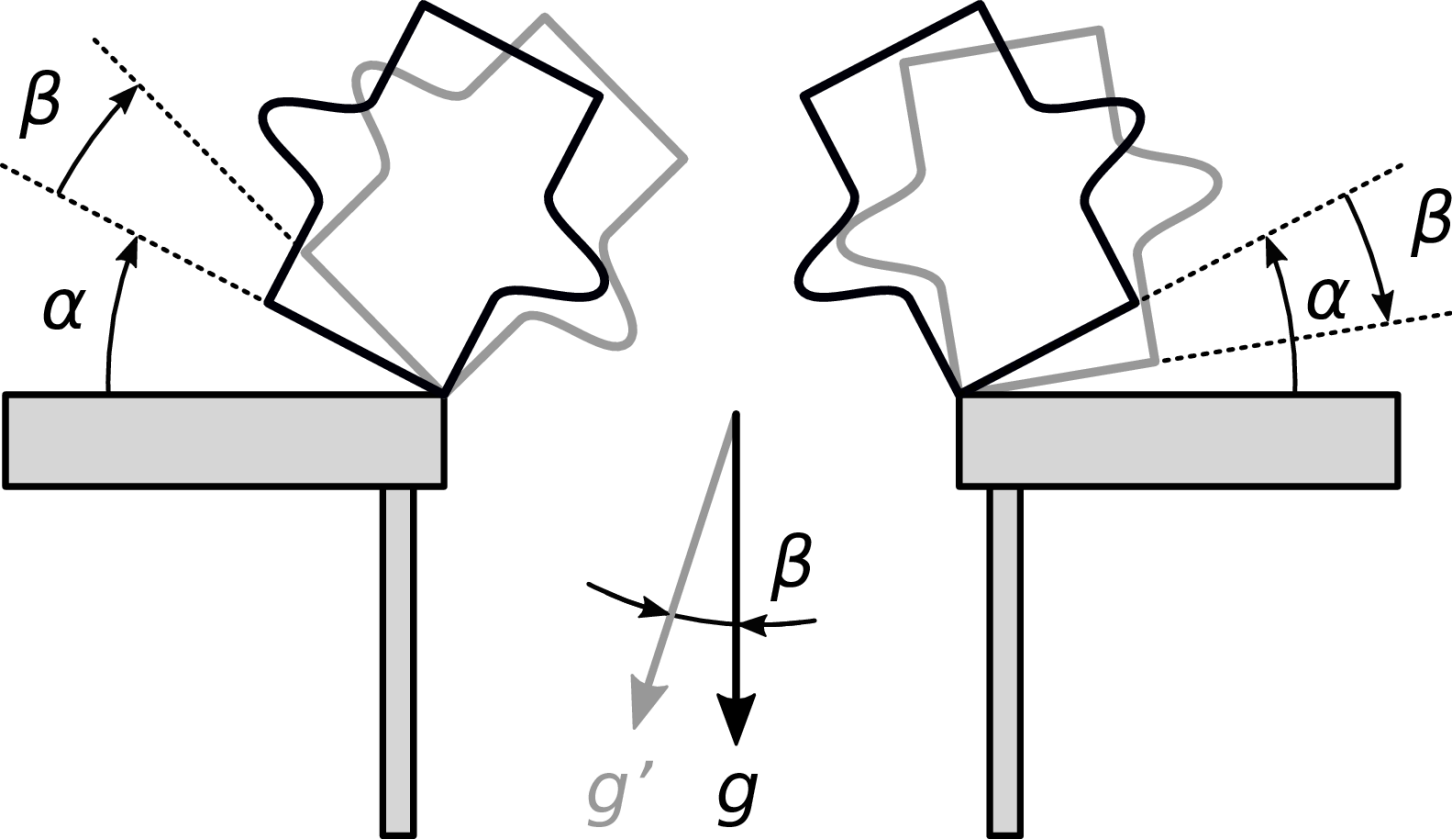
Theoretical predictions for the mirror-scene experiment. The hypothetical critical position for an object under normal condition (static background) are shown with black lines. The perceived gravity vector (*g)* is indicated. The critical angle for both left and right mirrored scenes is α. Under a perturbed condition, in which the perceived gravity vector (*g’*) is rotated clockwise by an angle β, the new theoretical critical positions of the object in both sides are shown with gray lines.

Experiment 1 showed that a moving visual scene induces an overestimation of the stability of falling objects. Whether the perceived CA changes in the same direction as perceived gravity, it remains unclear how both phenomena could be related. However, the deviation in the CA estimation induced by the rotating surround was smaller than one would predict when taking into account the deviation in the perceived gravity induced by vection. According to Samuel and Kerzel [19], this anticipative behavior (i.e. judging that the objects would fall, even though they would stay upright according to the laws of physics), may have been guided by a tendency to stay “on the safe side.” This conservative bias could be explained by difficulties in judging object stability. Experiment 2 was designed to explore to which extent an erroneous estimation of the gravity vector affects the object stability judgment. In addition to the scene configuration used in Experiment 1, we presented also the mirrored scene (the object and the table on the right side) to the participants. We hypothesize that, under vection, the right scene will be intrinsically less stable than the left scene because objects in the former case are tilted to the left and, hence, the line connecting their COM to the table edge get aligned with the perceived gravity direction earlier (see Fig 4 for a pictorial explanation). Furthermore, any conservative bias that may exist would be canceled out when computing the difference in the estimated CA between both scene sides. Hence, if the shift in CA estimation is directly related to the shift in the perceived gravity direction under vection, then the scene side effect on the CA should be twice the amount of the shift in TU.

### Methods

#### Participants

Sixteen new paid volunteers (7 females, 9 males, ages: 20–29 years, mean ± SD: 22.7 ± 2.7 years SD) with normal or corrected to normal vision and no history of vestibular dysfunction took part in Experiment 2. As in Experiment 1, participants were naive to the purpose of the experiment and provided written informed consent.

#### Stimuli

For the perception of the gravity direction, stimuli and method were the same as in Experiment 1. The visual surround was static or rotated rightward at an angular velocity of 30°/sec. For the CA estimation, two left/right symmetrical scene configurations were used (illustrated in Fig 4): either the object was tilted rightward over the precipitous right edge of the table or the object was tilted leftward over the precipitous left edge of the table. Everything else was the same as in Experiment 1.

#### Procedure

Participants, seated upright, performed 4 experiments in one session of one hour: TU estimation task with static and moving surrounds, CA estimation for rightward and leftward tilted objects with moving surround. A practice session of 20 stimuli, with static surround, was performed at the beginning of each task. All the participants began with the TU estimation task, the static and moving surround conditions being counterbalanced across them. Due to constraints on the duration of the experimental session, for the TU task, each subject was tested with the table on either the left or the right side. In the CA estimation task, rightward and leftward tilted objects were grouped in separate blocks. The order of presentation was counterbalanced across the participants.

#### Data analysis

Data analyses were essentially the same as in Experiment 1. When analysing the data for the CA estimation task, we used an LMM with two random effects. We tested the significance of these effects separately using the anova function for merMod objects of the lme4 R package [16] which computes the likelihood ratio between two models (one containing the random effect being tested and the other without it) and test it against the χ^2^ distribution. Random effects for each subject are estimated by the BLUP (best linear unbiased estimators) procedure, provided by the ranef function of the lme4 package.

### Results

**Fig 5:**
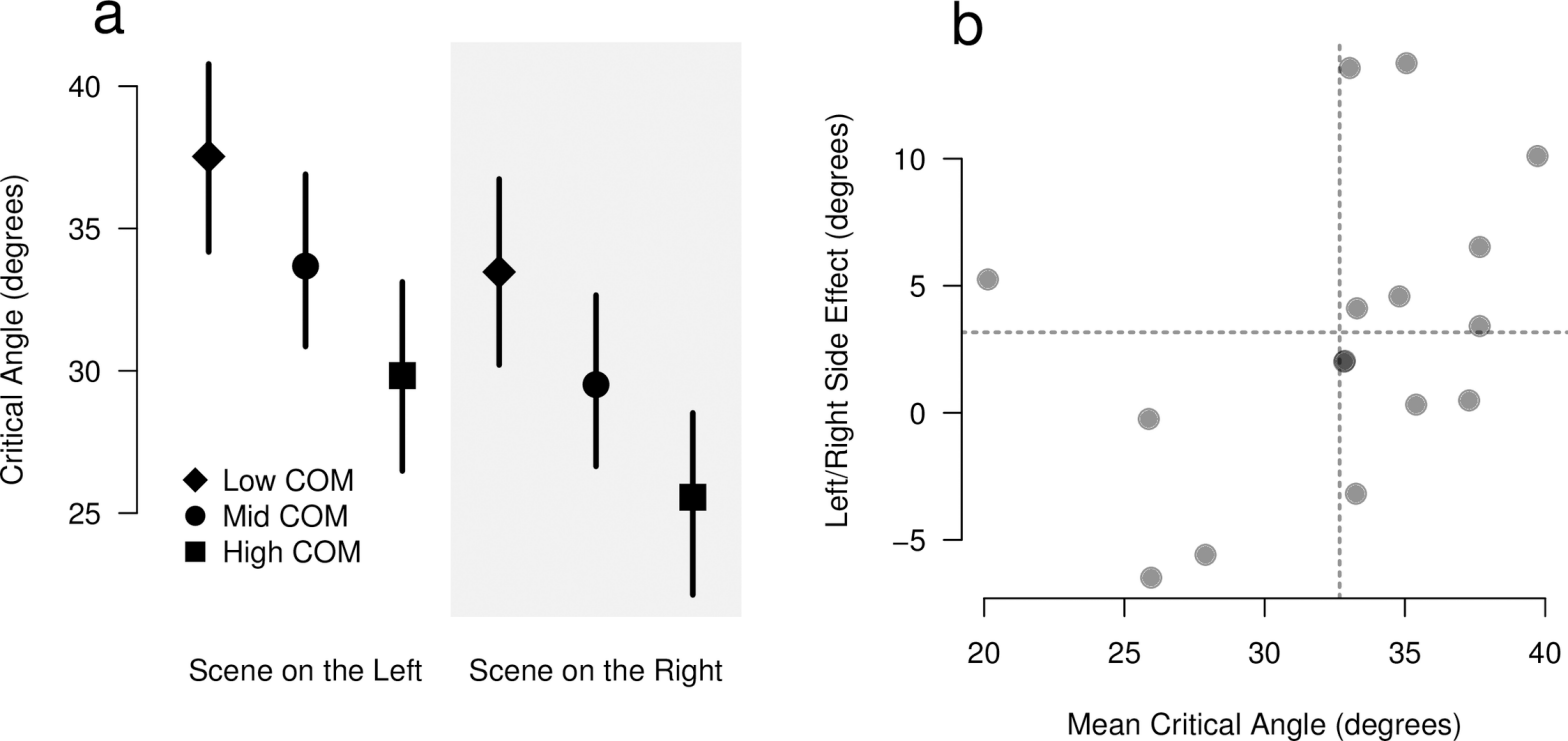
Results of Experiment 2. Panel a: Mean estimated critical angles for the scenes presented on the left and on the right sides of the display. Vertical lines indicate the 95% confidence intervals for each condition. Panel b: Random effects for the critical angle analysis. Each subject is represented by a dot. The estimated mean critical angle across sides and objects is shown in the horizontal dimension. The vertical dimension represents the per-subject estimated difference between the right and the left sides. The vertical and horizontal dotted lines represent, respectively, the global mean value for the CA and the mean estimated difference in CA across scene sides.

Results for the effect of scene side on the CA estimation under rotating background are shown in Fig 5A. There is a significant effect of the scene side on the estimated CA (F(1,15) = 5.50, p < 0.034). The difference in CA estimation between the left and the right sides was 4.16° (95% CI [0.58°, 7.75°]). As in Experiment 1, there was a significant effect of the COM height on the CA estimation (F(1,62) = 77.42, p < 0.001), with a difference of 3.85° (95% CI [2.62°, 5.08°]) for the low- and high-COM objects in comparison with the mid-COM object. No interaction effect between COM height and scene side was observed.

We fitted to the data an LMM with two random effects, namely an overall CA deviation for each participant and a dependency on the scene side for each participant. Both random effects were significant, with SD = 5.12° (χ^2^(1) = 56.97, p < 0.001) and SD = 6.49° (χ^2^(1) = 25.80, p < 0.001), for the intercept and scene side random effects, respectively. These results indicate that the inter-participant variability, both in terms of the intercept and the scene-side effects, was important in our experiment. The BLUP values for the random effects associated with each participant are shown in Fig 5B. Notice that, in this figure, the mean CA values estimated for the intercept and the scene-side fixed effects are added to the BLUP values. The subject who presented the highest scene-side effect has a right/left deviation in CA of 13.8°.

As regards the TU estimation, there is a marginally significant difference of 1.65° between the left and right scene sides (F(1,14) = 3.70, p > 0.074). A significant effect of the rotating/static background of 7.17° was found (F(1,14) = 64.34, p < 0.001). Notice that the individual right/left deviations range from -5.6° to 13.8°. The maximum value in this range is close to twice the TU shift, even though the population mean value (4.16°) is much lower than that.

## Experiment 3

In Experiment 2, the effect of the rotating background on CA estimation across scene sides was smaller than what would be predicted from the static vs. background effect on TU estimation. An essential difference between the CA and TU estimation tasks concerns the visual cues to orientation, i.e. the presence of a trial-to-trial stationary upright table during the former task. It has been shown that the cognitive polarity of objects can modulate the perception of orientation with respect to gravity [20, 21]. Typically, the components of a table are perceived as aligned with gravity (the feet) or perpendicular to it (the top). Hence, the presence of a stationary table could define a frame of reference for orientation and could explain why the rotating background affected less the CA estimation than the TU estimation. An additional experiment was run, in which participants estimated the CA with and without the table, in order to verify this hypothesis.

### Method

#### Participants

Seventeen new paid volunteers (10 females, 7 males, ages: 21–27 years, mean ± SD: 23.4 ± 1.6 years) with normal or corrected to normal vision and no history of vestibular dysfunction took part in Experiment 3. As in Experiment 1 and 2, participants were naive to the purpose of the experiment and provided written informed consent.

#### Stimuli

The stimuli for the CA estimation task were the same as in Experiment 1 but we added an extra condition where the rightward tilted object was presented without the table. The visual surround was either static or rotating clockwise at an angular velocity of 30°/sec.

#### Procedure

Participants, seated upright, performed 4 experimental blocks in two sessions of 40 minutes each: (1) CA estimation for tilted objects in presence or absence of the table with static surround, (2) CA estimation for tilted objects in presence or absence of the table with moving surround. A practice session of 20 stimuli was given at the beginning of each task with static surround. The order of the session (static vs. moving surround), as well as the order of the table condition (absent vs. present) during each session was counterbalanced across participants. The time interval between two sessions was at least two hours.

#### Data analysis

The statistical techniques used for analyzing the data were the same as in Experiments 1 and 2.

### Results

**Fig 6:**
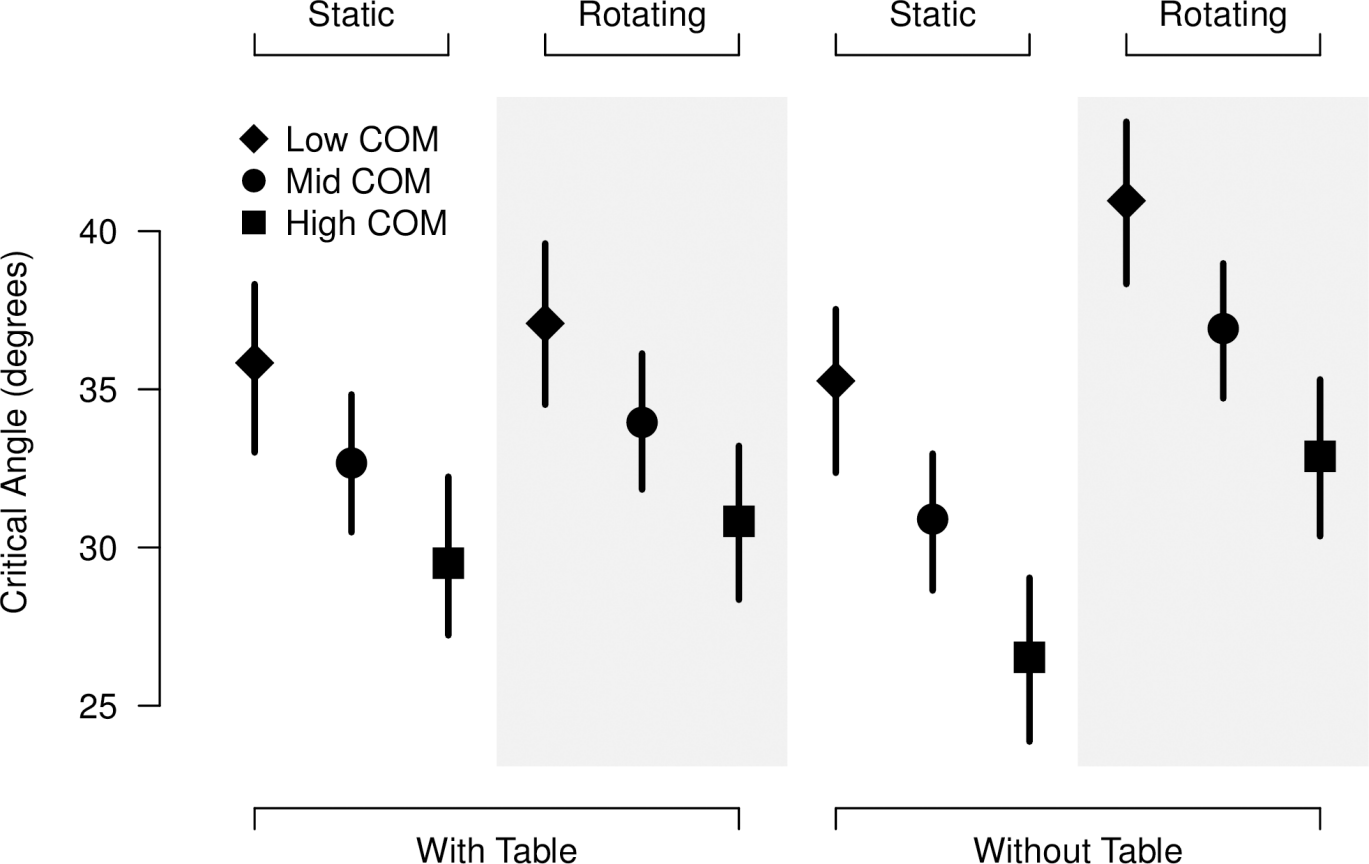
Results of Experiment 3. The values for the mean estimated critical angles are shown. The conditions where the table is present are grouped on the left side, while those when the table is absent are on the right side. The static and rotating background conditions are shown in the white and gray strips, respectively. Vertical lines indicate the 95% confidence intervals for each condition.

Results for Experiment 3 are shown in Fig 6. The data were fitted with an LMM model with three fixed factors: the presence of the table, the movement of the surround (static vs. rotating) and the COM height (low, mid, and hi). All interaction factors among the fixed factors were also considered in the model. A random factor allowing a deviation in the intercept by participant was also included. As in Experiments 1 and 2, there was a significant effect of the COM height on the CA estimation (F(1,180) = 115.22, p < 0.001), with a difference of 3.17° (95% CI [1.84°, 4.49°]) for the low- and high-COM objects in comparison with the mid-COM object. The surround factor showed a significant effect (F(1,180) = 42.70, p < 0.001), which was not the case for the presence/absence of the table. However, the interaction factor table × surround had a significant effect (F(1,180) = 17.96, p < 0.001). The difference in the estimated CA between the static and rotating surrounds is higher without the table than with it (4.74°, with 95% CI [2.58°, 6.90°]). The other interaction factors had no significant effects.

## Discussion

### Influence of vection on object stability judgment: General findings

Across three experiments, we investigated whether bias in the perceived orientation of gravity induced by visual motion influenced the judgment of object stability. In line with previous experiments, we observed overall that participants estimated the critical angle for the fall off of objects in a manner that is coherent with the physics of the situation, i.e. by taking into account the object’s COM height. In the moving surround condition, the estimated CA was deviated toward the direction of the perceived gravity, as indirectly measured with the TU estimation task. However, the average amount of deviation of CA estimation under vection was smaller, in average, than the amount of deviation in the perceived gravity. Moreover, when analyzing the individual results, no significant correlation between these two deviations was found. Thus, in a vection situation, the perceived gravity does not seem to be the major cause of deviation in object stability judgment. Finally, results of Experiment 3 suggested that the presence of the table in the scene significantly reduced the effect of the moving surround on the CA estimation.

### Reference frames used in CA estimation

Perception of space depends on reference frames from which judgments of position, orientation, and motion are derived [22, 23]. The visual environment is richly endowed with information that can be used to derive spatial orientation with respect to gravity [24], such as sets of lines and surfaces which are normally aligned with or orthogonal to gravity, and visual polarity of objects which show an identifiable "up" and "down" [20, 21, 25]. It can be suggested that the table in our experiments may provide a reference frame for estimating the direction of gravity. Barnett-Cowan et al. [1] measured the effect of body tilt on the perceived verticality of a visual rod in the presence or absence of an upright table. Even though the presence of the table decreased the Aubert effect observed in the situation where the table was absent, it must be noted that the visual reference frame did not suppress this effect completely.

In a similar way, the presence of the table decreased the effect of the moving visual surround on the judgment of object stability. However, it must be emphasized that the presence of stationary objects in a scene, like the table in our experiment, should not affect the amount of vection illusion per se [26]. This suggests that both the body tilt induced by the moving surround and the visual reference frame provided by the table contribute to the perception of gravity, that is needed in the CA estimation task. This finding is true at the population level, even though participants differently weigh both visual orientation cues when estimating the CA.

### Real and simulated tilt have different influences on the CA estimation

In the real tilt situation, the orientation of the body had no significant effect on the estimated CA. Barnett-Cowan et al. [1] found an average effect of 15.0° for the visual deviation of the direction of gravity with 90° body tilt, that is close to 3 times the effect we found with 30° of body tilt (4.45°). However, they found a change in the CA estimation with real body tilt, which is not the case in our experiment. One possible explanation for this difference may come from the nature of the stimuli (3D perspective representation in their study and pure 2D in ours).

Both vection and body tilt caused a displacement of the perceived gravity direction. Notice that the amount of TU change caused by vection was not significantly different from that caused by the 30° body tilt (95% CIs [2.50°, 4.34°] and [2.80°, 6.11°], respectively). However, vection influenced the CA estimation, as discussed above, while real body tilt did not. One possible reason for this difference is that the visuo-vestibular conflict caused by the rotating surround has the potential to induce disorientation. It has been suggested that when disorientation occurs, there is a natural tendency to revoke resources allocated to secondary tasks and redirect them to the reestablishment of orientation [27]. This resource reallocation has been shown to effectively affect the performance of secondary tasks [28]. Finally, it can be suggested that the presence of the table influences the CA estimation in different ways for real and simulated body tilt. This difference can be explained by the fact that, in the rotating surround condition, the subject perceived the top-down axis of the table aligned with his/her body axis and had the illusion that his/her own body is tilted. Hence, from the subject’s perspective, the table cannot be in the upright orientation and, therefore, the reference frame provided by the table may be less reliable in the simulated tilt situation than it is in the real tilt situation.

### Implications for virtual reality usage

Vection is of practical relevance because it is crucial for making vicarious virtual reality displays by providing a compelling experience of “presence” in such environments. However, the present study showed that a moving visual environment biases spatial estimates and to some extent expectation and predictive behavior of objects. Thus, visually-induced distortions of spatial orientation may be critical for the perceptual guidance of motor actions in simulated environments.

### Limitations of the present study

This study has some limitations, though. In the literature, studies of induced rotation have provided important contributions to the understanding of visually induced motion, but it must be recognized that rotation about the line of sight (roll) occurs less frequently than translation or rotation around the vertical axis (yaw) in real and virtual environments. Moreover, it has been shown that attentional load could weaken vection [29]. It may be the case that the subjective strength of vection is smaller in the object stability task in comparison with the table uprightness task, because the former requires attentional resources related to spatial perception. Note that the objective measurement of the subjective amount of vection is not easy to accomplish without more elaborated neurophysiological techniques.

## Supporting information

S1 Video Example of stimulus display. In this video, an example of the experimental display is shown, for the rotating surround condition and the critical angle estimation task. The outer circle represents the area seen by the subject, whose visual angle is 50°. Only ten seconds of the initial, habituation rotation period is shown (in the experiment, this period took 60 seconds). The eleven first initial trials are shown (each block contained 120 trials) and they are displayed in the same timing (stimulus duration and inter-stimuli interval) and as it was done in the experiments.
(MP4)

S1 Text Theoretical critical angles (CA) for the objects used in the experiments. In this document, we derive the theoretical values for the CA of the objects used in our study, based on the laws of Physics.
(PDF)
