# Supplementary material for: Real and visually-induced body inclination differently affect the perception of object stability
Source: PLoS One. 2017 Oct 16;12(10):e0186431. doi: 10.1371/journal.pone.0186431 (PMC5643064; doi:10.1371/journal.pone.0186431)
Supplement: S1 Text — In this document, we derive the theoretical values for the CA of the objects used in our study, based on the laws of Physics. (PDF) [file pone.0186431.s002.pdf]

Supplemental material for “Real and visually-induced body inclination differently affect the perception of object stability”

Rafael Laboissière, Pierre-Alain Barraud, and Corinne Cian

May 2017

In the present document, we derive the theoretical values for the critical angles (CA) of the objects used in our study, based on the laws of Physics.

The geometry of the objects used in the experiment is depicted in Fig. S1. In the actual stimuli, the lateral contour of the bulged parts was generated with Bézier curves. Here, we approximate them with straight lines, such that the bulged parts look triangular.

The values of the geometric parameters used in the experiments are (in arbitrary units):

$$\begin{aligned} r &= 32.5 \\ h &= 110 \\ a &= 40 \\ b &= 30 \end{aligned}$$

The value of c varies according to the object type. Its relation with parameters a and h is shown in the right panel of Fig. S1.

In our experiments, subjects were instructed to consider the scene as representing a real 3D scene projected onto the 2D screen. In order to compute the center of mass (COM), assumptions must be made regarding how the 3D shape of the objects is derived from the 2D projection. In Fig. S2, we represent two possible ways of doing it. In the first interpretation, the object would have a cylindrical symmetry while, in the second, the object would be flat with a certain depth.

In the cylindrical symmetry case, the object can be decomposed into two parts: a central cylinder and a triangular ring. Assuming an homogeneous material with density ρ, the mass of both parts and the

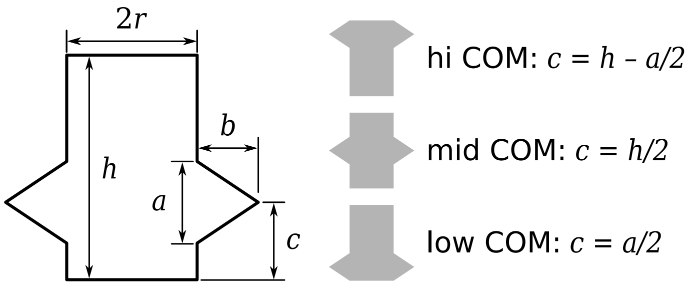

Figure S1 – Schematic representation of the object used in the stability perception study. Left panel: Geometry of the generic object with indication of the geometric parameters. Right panel: The three types of objects used in the experiment, along with the algebraic relations among the geometric parameters.

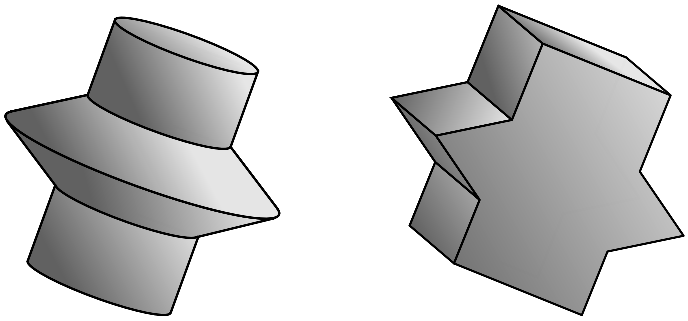

Figure S2 – Two possible 3D representations of the object used in the experiments. Left: cylindrical symmetry hypothesis. Right: flat object hypothesis.

total mass of the object are:

$$
\begin{aligned}
m_{\text{central}} &= \rho h \pi r^2 \\
m_{\text{ring}} &= 2\rho\pi(r+b/3)ab/2 \\
m &= m_{\text{central}} + m_{\text{ring}}
\end{aligned}
$$

The vertical positions of the COM for each object in the upright position are computed as a weighted sum of the vertical position of the COM of the parts:

$$
\begin{aligned}
y_{\text{LOW}} &= [(h/2)m_{\text{central}} + (a/2)m_{\text{ring}}]/m \\
y_{\text{MID}} &= [(h/2)m_{\text{central}} + (h/2)m_{\text{ring}}]/m \\
y_{\text{HIGH}} &= [(h/2)m_{\text{central}} + (h-a/2)m_{\text{ring}}]/m
\end{aligned}
$$

The critical positions of the objects are shown in Fig. S3. The CAs can be computed as:

$$
\begin{aligned}
\text{CA}_{\text{LOW}} &= \arctan(y_{\text{LOW}}/r) \\
\text{CA}_{\text{MID}} &= \arctan(y_{\text{MID}}/r) \\
\text{CA}_{\text{HIGH}} &= \arctan(y_{\text{HIGH}}/r)
\end{aligned}
$$

For the case where the object is interpreted as being a solid plate, the masses of the parts (the central rectangle and teh sum of the two lateral triangles) are given by:

$$
\begin{aligned}
m_{\text{central}} &= \rho 2rh \\
m_{\text{ring}} &= \rho ab
\end{aligned}
$$

The equations for computing CA_{LOW}, CA_{MID}, and CA_{HIGH} are the same as in the cylindrical symmetry case.

The table below shows the numerical values of the CA for each object and for both hypothesized shapes, as well as the results for Exp. 1 (upright body orientation, static surround). The estimated values and the 95% confidence intervals for the experiment are reported.

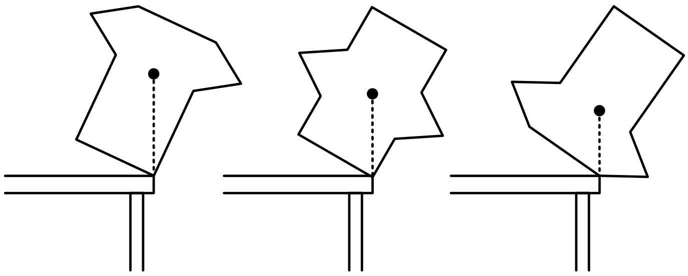

Figure S3 – Critical positions for the high-, mid-, and low-COM objects.

COM position	cylinder	plate	Exp. 1
low	36.2°	33.0°	35.4° [33.6°, 37.2°]
mid	30.6°	30.6°	32.1° [30.3°, 33.9°]
high	26.3°	28.4°	28.7° [26.8°, 30.5°]

We notice that the confidence interval obtained in our experiment include the values of the solid plate hypothesis, even though the values for the cylindrical symmetry hypothesis are not too far off.
